# Psoriasis

**Published:** 1885-10-20

**Authors:** 


**Psoriasis.**—Dr. F. proposes for psoriasis the following prescription :

℞. Chrysarobin . . . . . . . . . . . . . . . . . . . . . . . . . . . 10 parts

Salicylic acid . . . . . . . . . . . . . . . . . . . . . . . . . . . 10 parts

Ether . . . . . . . . . . . . . . . . . . . . . . . . . . . . . . . . 15 parts

Flexible collodion to . . . . . . . . . . . . . . . . . . . . . . 100 parts

This combination does not produce the staining of the skin, injury to the clothing, and in some instances, the dermatitis, which most of those who have used the chrysophanic acid must have observed.

In acne vulgaris, Dr. Piffard, of N. Y., recommends the bromide of arsenic in 1–100—1–50 grain doses, twice or three times a day.

In eczema marginatum and in ringworm in general, Dr. R. W. Taylor, of N. Y., recommends a solution of the bi-chloride of mercury in the compound tincture of benzoin, or any gum reisin, in the proportion of two to four grains to the ounce. The resin holds the bi-chloride in contact with the diseased skin.

In eczema—reported in connection with a case of eczema of leg—Dr. Morrow, of N. Y., suggests a gelatin plaster, and eight of water, medicated with ten per cent. of oxide of zinc and one per cent. of carbolic acid. This plaster may be spread on muslin and evenly applied to the inequalities of the surface.—*Ex.*

